# Exploring stroke survivor and employer experiences of disruption within the RETurn to work After stroKE (RETAKE) trial during the COVID-19 pandemic

**DOI:** 10.3389/fsoc.2025.1434353

**Published:** 2025-07-02

**Authors:** Diane Trusson, Katie Powers, Kate Radford, Audrey Bowen, Kristelle Craven, Amanda Farrin, Christopher McKevitt, John Murray, Julie Phillips, Judith Stevens, David Clarke

**Affiliations:** ^1^School of Medicine, University of Nottingham, Nottingham, United Kingdom; ^2^NIHR Nottingham Biomedical Research Centre, Nottingham University Hospitals NHS Trust, Nottingham, United Kingdom; ^3^School of Medicine, University of Leeds, Leeds, United Kingdom; ^4^Clinical Trials Research Unit, University of Leeds, Leeds, United Kingdom; ^5^Social Sciences and Health, King’s College London, London, United Kingdom; ^6^Patient and Public Involvement (PPI), University of Leeds, Leeds, United Kingdom

**Keywords:** stroke, return-to-work, biographical disruption, normalisation process theory, vocational rehabilitation, COVID-19 pandemic, interviews, United Kingdom

## Abstract

**Introduction:**

Returning to work is a goal for many stroke survivors, with benefits for individuals and society. The ReTurn to work After stroKE (RETAKE) trial, which aimed to improve stroke survivors’ work outcomes through early stroke-specific vocational rehabilitation (ESSVR), was ongoing during the COVID-19 pandemic. This study aimed to understand the impact of the pandemic on stroke survivors’ work ability and return-to work support.

**Methods:**

Nine stroke survivors and five employers were interviewed. Thematic analysis informed by Normalisation Process Theory, found that biographical disruption experienced as a result of stroke was compounded by disruption on a global scale due to the pandemic.

**Results:**

Attempts to mobilise resources in response to disruption were hampered by pandemic-related issues. Although returning to work offered continuity in pre-stroke identity, businesses were also disrupted by the pandemic. Findings suggest that returning to work was easier for stroke survivors able to work from home and those receiving ESSVR. The opportunity to work from home helped stroke survivors adapt to new ways of working necessitated by the impact of stroke and social distancing rules during the pandemic.

**Discussion:**

Post-pandemic, remote working is more acceptable, which may benefit future stroke survivors aiming to return to work whilst managing post-stroke fatigue. This may mitigate disruption to lives and post-stroke identities.

## 1 Introduction

Stroke is a leading cause of death and disability worldwide. In the UK alone, approximately 110,000 people experience stroke each year, (Stroke Association, 2025) and although around 25% of stroke survivors are of working age, only about half return to work (RTW) following stroke (Daniel et al., 2009; Edwards et al., 2018). A 2022/23 survey reported that the proportion of patients working full-time 6 months after stroke was 6.1% compared to 14.5% in full-time employment before stroke (Stroke Association, 2023). As well as the financial benefits associated with employment, previous studies indicate that returning to- and staying in work are associated with improved mental and physical health (where work provides security and a level of personal control; Langhammer et al., 2018). Conversely, being unemployed can negatively impact health and wellbeing, both physically and mentally (Daniel et al., 2009). Previous studies have identified several significant predictors of RTW after stroke, including less severe stroke, greater independence in activities of daily living, having a ‘white-collar’ or ‘qualified worker’ job, better cognitive ability, and fewer neurological deficits (Ashley et al., 2019; Edwards et al., 2018). Given the importance of RTW following stroke, and the number of factors that can impact this, further exploration of how these factors interact and facilitate or prevent RTW within the context of the pandemic is warranted.

Vocational rehabilitation (VR) is an intervention that supports someone to stay in, or RTW (Waddell et al., 2008). Although VR is considered a key element of successfully achieving and sustaining RTW following stroke (Baldwin and Brusco, 2011), there is a lack of robust empirical evidence to support this. The RETurn to work After stroKE (RETAKE) trial was developed to address this gap (Radford et al., 2020; NHS, 2021; Powell et al., 2022). RETAKE was a large multi-centre randomised controlled trial testing the clinical and cost-effectiveness of an early stroke specific vocational rehabilitation (ESSVR) intervention compared to usual care only.

Usual care varies across the UK but typically involves NHS rehabilitation which may include outpatient/community physiotherapy, speech therapy, occupational therapy, psychology and medical follow-up. However, an audit of post-acute stroke care provision reported that these services are often missing or patchy, with just 39% of patients having an assessment of their needs 6-months after their stroke (King’s College London, 2024). The authors concluded that geographical variation in provision needs to be eradicated and recognized that a wider group of stroke patients require ongoing rehabilitation. In addition, only 25% of services were commissioned to deliver VR (King’s College London, 2021). A 2022/23 survey found that 90% of stroke survivors employed or self-employed before their stroke agreed that RTW was important to them, but only 28% agreed that their therapy team provided RTW support (Stroke Association, 2023).

The ESSVR intervention was developed to address gaps in current provision (Sinclair et al., 2014). ESSVR started soon after stroke onset and was delivered alongside usual care by an occupational therapist (OT) with specialist stroke knowledge and VR training who provided tailored support to stroke survivors for up to 1 year. The aim was to support stroke survivors to RTW and remain in work. Table 1 summarises ESSVR:

**Table 1:** Summary of ESSVR.

Early Stroke Specialist Vocational Rehabilitation (ESSVR) Complex, individually tailored, manualised intervention that adopts a case co-ordination model. Delivered by occupational therapists to stroke survivors, their employers, and their families. Provides a re-accessible service for up to 12 months post stroke. Core components include: Early intervention (within 12 weeks of stroke, providing early advice on impact of stroke & RTW) Assessing impact of stroke on person/family & job (analysing work ability, worksite assessment) Delivering individually tailored vocational rehabilitation (Work preparation, RTW planning) Communicating openly in writing with stakeholders regarding work status. Co-ordinating vocational rehabilitation across all health, social, and employment sectors. Providing education, advice and emotional support to stroke survivor, family and employer. Mediating workplace adjustments, negotiating phased RTW, and providing feedback on performance. Monitoring RTW to ensure work sustainability (regular review, supporting employer to provide feedback on work performance, and feeding back on progress and modification). Exploring alternatives where current work cannot be sustained or is not feasible. Gradual withdrawal of the intervention, which the stroke survivor can re-access, as required, up to 12-months post-randomisation.

Further details of the RETAKE trial and ESSVR are given in Radford et al. (2020, 2024), Radford (2025) and Trusson et al. (2025) in the logic model (Appendix 1). An embedded process evaluation gathered data from multiple sources including stroke survivors, their nominated carers, OTs, and employers. The aim was to examine fidelity to ESSVR and explore the social and structural factors that may have influenced implementation in the trial (Radford et al., 2020). However, the COVID-19 pandemic (henceforth ‘the pandemic’) occurred whilst the RETAKE trial was in progress, affecting opportunities and processes for supporting RTW.

At the start of the pandemic, to minimise the spread of COVID-19, the World Health Organisation (WHO) recommended social distancing and stay-at-home measures with people told to work from home where possible. The United Kingdom (UK) government introduced the Coronavirus Job Retention Scheme spanning 18 months (March 2020 to September 2021) (Gov.UK, 2020a) which provided grants to enable employers to furlough their employees at up to 80% of their wages (Powell et al., 2022). During the pandemic, NHS services were heavily impacted, leading to government directives to ‘protect the NHS’. People considered clinically vulnerable received support to help them remain indoors (HM Government, 2020). NHS staff were redeployed to help treat COVID patients, affecting routine (non-urgent) procedures, and causing staffing shortages in many NHS services, including stroke rehabilitation.

In the RETAKE process evaluation, the pandemic resulted in fewer opportunities to interview employers (Clarke et al., 2024). Also, the importance of capturing stroke survivors’ (and employers’) experiences in the context of the pandemic was recognised. This study, following on from the process evaluation, aimed to understand the impact of the pandemic on stroke survivors’ work ability and RTW support from the perspectives of stroke survivors and employers (Radford et al., 2022).

## 2 Method

This study used qualitative methodology taking a broadly interpretivist approach to ‘understand people’s perspectives in the context of the conditions and circumstances of their lives’ (Ormston et al., 2014, p. 22). Semi-structured interviews were used to explore in detail how stroke survivors and employers perceived the impact of the pandemic on post-stroke RTW support (Yeo et al., 2014; Radford et al., 2022). Similarly to the RETAKE process evaluation, Normalisation Process Theory (NPT) informed data collection and analysis (Clarke et al., 2024). Whilst NPT is often used to understand healthcare professionals’ experiences of implementing new technologies or techniques (May et al., 2009),in this study, as in the RETAKE process evaluation, NPT was used as a sensitising lens when analysing stroke survivors’ experiences (Clarke et al., 2024). Previous studies have used NPT to explore treatment burden in patients, including stroke survivors, where the four NPT constructs [coherence, cognitive participation, collective action, and reflexive monitoring (May et al., 2009)] were reinterpreted as ‘sense making; interacting with others; enacting management strategies; and appraisal work’ (Gallacher et al., 2013, p. 2). These terms informed the current study’s analysis.

### 2.1 Participants

After gaining ethical approval, potential participants were e-mailed study details. Stroke survivors were eligible for this study if they were RETAKE trial participants, had consented for contact about future studies, and had experienced stroke during or shortly before the pandemic. We aimed for a gender balance to ensure that the female perspective was represented (Ibrahim et al., 2024). We also selected self-employed participants and those employed in industries most adversely impacted by the pandemic (e.g., leisure, catering, and service industries).

Stroke survivor participants (who were not self-employed) were asked for their consent to approach their employer for interview. Previous research has highlighted the difficulties in recruiting employers (Coole et al., 2013), therefore, we also sought to recruit employers using established clinical academic links and partnerships with employers and industry.

### 2.2 Data collection

Semi-structured telephone or video-conference interviews with stroke survivors and employers were conducted by the first two authors. The topic guides were developed with reference to NPT (Appendix 2). Verbal consent was recorded at the outset of each interview, with participants assured of the confidentiality of their responses. Employer participants were reminded not to reveal any personal information about the employee they were discussing. Interviews were digitally recorded (with permission), and professionally transcribed.

### 2.3 Analysis

**Figure 1:**
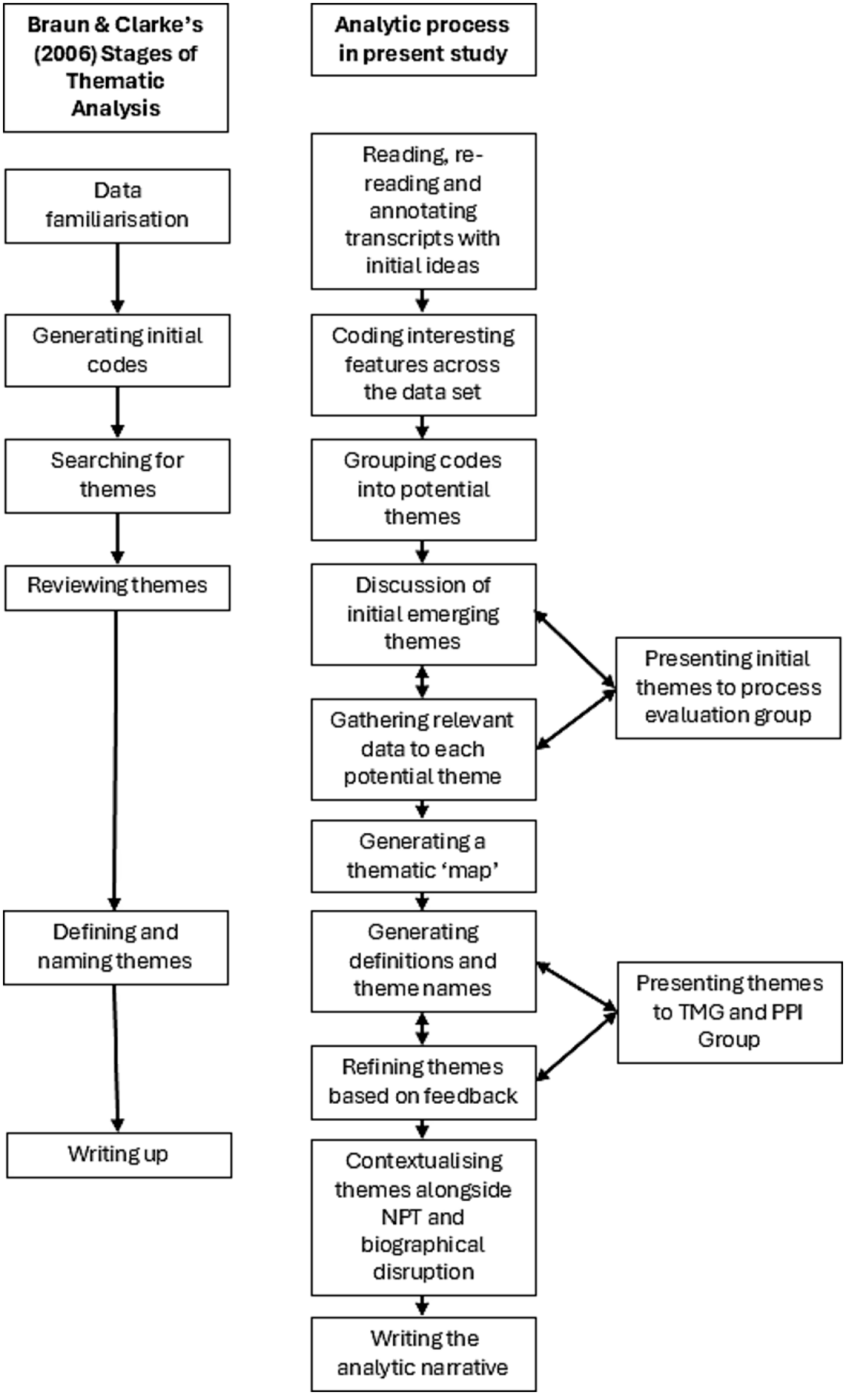
Analytic process.

A systematic approach to thematic analysis was used, informed by the phases recommended by Braun and Clarke (2006). The first two authors read the transcripts multiple times to familiarise themselves with the interview data, examining the transcripts line-by-line and highlighting quotes. They analysed the data independently, examining emerging themes to determine the extent to which they addressed the key study questions whilst remaining open to novel and unexpected findings. The emerging themes were discussed and agreed by the lead authors once the independent review of interview data was completed. Themes and the ongoing analytical approach were then shared with the wider study team. Figure 1 illustrates the analytic process.

The initial inductive approach ensured that coding and theme development were data-led. A subsequent deductive approach involved mapping the themes onto the reinterpreted NPT constructs and components (see Table 2).

**Table 2:** NPT constructs applied to interview data.

NPT constructs/ components	Barriers experienced	Facilitating experiences
Coherence: ‘Sense making’ (A need to understand the impact of stroke and implications for RTW) Differentiation	Reluctance to further burden the NHS when health services were under strain due to the pandemic. Worry about recurrence due to lack of knowledge about possible cause of stroke. Anxiety about wasting their GPs’ time on what they considered to be trivial matters in comparison to the needs of more seriously ill patients. (Differentiation)	RETAKE OTs available for advice, guidance and answering ‘silly’ questions which eased participants anxiety. Being part of the trial legitimised their use of resources.
Cognitive participation: ‘Interacting with others’ (Having the knowledge, skills and resources to adapt to post-stroke abilities) Legitimation	Information was perceived as too vague. Reluctance to disclose the (full) impact of stroke to family, employers, work colleagues etc. (lack of enrolment)	Facilitated where employers and colleagues were willing to be educated about stroke. Provision of advice and guidance, including written reports for stroke survivors and employers, by OTs with specialist knowledge in VR and stroke (Legitimation)
Collective action: ‘Enacting management strategies’ (Facilitating adaptations to post-stroke abilities) Interactional workability Skill set workability	Collective action hampered by cessation of community stroke rehabilitation support after 6–8 weeks. Lack of RTW advice/support. Reluctance to accept adjustments. Businesses closed during pandemic. Delays in receiving documentation from GP	OT’s knowledge and experience helped participants to know their limitations and suggest strategies, e.g., for managing fatigue. (Interactional workability) RETAKE OTs advocated in interactions with GPs and employers. OTs and/or OH services arranging phased return, workplace adaptations, adjusted hours, etc. (Skillset workability) New ways of working, e.g., working from home, online meetings
Reflexive monitoring: ‘Appraisal work’ (Seeking to understand ways that stroke affects themselves and others) Individual appraisal Reconfiguration	Stroke experienced as disruption to identity, e.g., work identity, and to future plans including work. For instance, retirement due to being unable to perform a role post-stroke. Wanting to know why stroke happened (meaning as consequence—Bury, 1982) Reflecting on what they might do to avoid another stroke (meaning as significance—Bury, 1982).	Plans to make positive changes, e.g., by changing job or role, working part-time or retiring (reconfiguration) Perception of RTW as a step towards restoring ‘normality’ and continuing identity Renewed zest for life; a ‘wake-up call’

Adapted from Gallacher et al. (2013).

Emerging themes were reviewed against the entire data set, which revealed a strong emergence of an overarching theme of ‘disruption’ in a range of areas. Consequently, it was decided that Bury’s (1982) theory of biographical disruption should be used to further examine and interrogate the data in order to gain insight into participants’ experiences of disruption resulting from both stroke and the pandemic.

The wider research team were consulted multiple times during the analysis process as the themes were reviewed and refined (see Figure 1). The study team included experts in stroke rehabilitation, occupational therapy and VR, as well as experts by experience. These public and patient involvement (PPI) members who had all experienced stroke whilst employed, were able to advise whether the themes resonated with their own experiences. This process of developing, reviewing and refining themes with input from a team of experts was designed to ensure trustworthiness in the analysis (Spencer et al., 2014). These processes underpinned our decision that sufficient data had been generated to address the study questions and led to the development of a robust and theoretically based explanation of the findings.

#### 2.3.1 Theoretical framework: biographical disruption

Bury’s (1982) concept of biographical disruption captures the disruption to every aspect of life (bodily function, relationships, everyday activities including work) and future plans, resulting from an unexpected diagnosis or illness event. Studies have highlighted the disruption to identities, roles, and relationships of working-age adults due to stroke (Kuluski et al., 2014; Wolfenden and Grace, 2015). For example, Wolfenden and Grace’s (2015) small sample of young stroke survivors experienced stroke as threatening to their identity, both as a personal sense of self, and how others perceived them. RTW was an important way of re-establishing and continuing their identity, despite the biographical disruption resulting from stroke.

Bury’s (1982) theory also explores how people bring meaning to disruptive experiences in two ways. Firstly, meaning as consequence (why it might have happened) and secondly, meaning as significance (what it might mean for the future). These issues were pertinent when considering and reporting on experiences of stroke and work in the context of societal-level disruption caused by the pandemic. Both types of disruptive experiences can be termed ‘critical situations’ (Giddens, 1979) in that, ‘structures of everyday life and the forms of knowledge that underpin them are radically disturbed’ (Bury, 1982, p. 169).

## 3 Results

### 3.1 Stroke survivor interviews

Nine stroke survivors (56% female) were interviewed. They were employed in various roles across the public (n = 5) and private (n = 2) sectors. In addition, two participants were self-employed. Table 3 reports demographic data.

**Table 3:** Stroke survivor participants.

Participant pseudonym	Gender & age	Employment sector	Trial arm*	Employer Interview?	Notes
‘Jackie’	Female 59	Part-time NHS Primary Care and part-time charity	ESSVR + UC	Yes	Phased RTW 6 months post-stroke
‘Lynn’	Female 63	Civil service	UC	Consent to contact employer not given	Phased RTW after ‘a few months’
‘Nick’	Male 58	Civil service	ESSVR + UC	Yes	No RTW. Seeking retirement on medical grounds
‘Wendy’	Female 59	Education	UC	Self-employed	RTW very soon after stroke
‘Graham’	Male 52	Higher education	ESSVR + UC	Employer did not consent to interview	Phased RTW after ‘a few months’
‘Emma’	Female 59	Personal care	ESSVR + UC	Self-employed	Phased RTW 3 weeks post-stroke
‘Chris’	Male 51	Steel industry	UC	Yes	Phased RTW 4 weeks post-stroke
‘Tess’	Female 45	Retail (food)	ESSVR + UC	Consent to contact employer not given	Phased RTW 4 months post-stroke
‘Liam’	Male 46	Local government	ESSVR + UC	Consent given to contact employer. Employer did not respond.	Phased RTW 4 months post-stroke

Note: When stroke survivor participants were recruited and interviewed it was not known who had, or had not, received ESSVR.

### 3.2 Employer interviews

Five stroke survivors gave permission to approach their employers. Of these, three employers gave consent and were interviewed. Additionally, an employer with experience of supporting a stroke survivor to RTW and an HR consultant with experience of supporting someone to RTW after a traumatic brain injury were recruited through established clinical academic links and partnerships with employers and industry. See Table 4 for details.

**Table 4:** Employer participants.

Employer participant	Type of employment	Notes
Employer A	Patient advocacy charity	Employer of a stroke survivor
Employer B	HR consultant	Experience of supporting employers where employee had traumatic brain injury
Employer C	Health-related charity	Employer of a stroke survivor
Employer D	Manufacturer	Employer of a stroke survivor
Employer E	Civil service	Employer of a stroke survivor

### 3.3 Themes

Analysis of the stroke survivor and employer interviews through the lens of biographical disruption (Bury, 1982) was particularly useful for exploring the experiences reported by stroke survivors and to a lesser extent when examining employers’ experiences. The themes and subthemes agreed by the study team are as follows:

1. Impact of the pandemic
2. Disruption to service provision, quality, and uptake
3. Disruption to working patterns and practices
4. Disruption to domestic life
5. Disruption to identity: (i) Work identity (RTW experiences; no RTW); (ii) Domestic identity and caring responsibilities; (iii) Identity as fit and healthy.

These themes and subthemes are discussed below, with pseudonyms used to protect participants’ identities.

### 3.4 Theme 1: impact of the pandemic

Stroke survivors reported myriad ways the pandemic had impacted on their lives, including disruptions in accessing and receiving NHS services and disrupted work and home lives. Stroke was experienced as a further disruption, impacting on identity both inside and outside the home, with consequent psychological impacts exacerbated by the pandemic.

In the following discussion of the findings, we draw on the concept of biographical disruption (Bury, 1982) in exploring both the stroke survivors’ and employers’ experiences of supporting RTW in the context of the pandemic. Stroke survivor participants are referred to by their pseudonyms.

#### 3.4.1 Disruption to service provision, quality, and uptake

Some stroke survivors experienced delays in accessing emergency services at the time of their stroke. They attributed delays to the pandemic:

I think the whole of the NHS is just really up against it at the moment, hence waiting 2 h for an ambulance when you have had a stroke (Wendy).

Some participants delayed seeking medical assistance because they were reluctant to further burden the NHS during the pandemic. They alluded to the government directive to ‘Protect the NHS’:

People said do not go into hospital if you do not think it’s serious and I did not know what it was—I thought it was just a dead leg (Lynn).

Once discharged from hospital, stroke survivors experienced delays within community services. Some participants reported that they did not receive any support from NHS services post-discharge:

Well, there was no—because of COVID, there was no physiotherapy, there was no help really, was there? (Lynn).

Where community services were accessed by stroke survivors, they reported reduced service provision and perceived quality (e.g., rehabilitation offered by phone or online rather than face-to-face).

There were also difficulties getting GP appointments. With a fear of putting a greater strain on resources some stroke survivors avoided contacting them:

Because of the current climate, you cannot get to the doctor’s, you feel really stupid going […] you cannot phone up the doctor and, you know, ask really silly questions, if you like (Tess).

Some participants seemed to have been discharged from usual care once a physical recovery had been achieved, without consideration for ongoing psychological issues:

I think the answer to all of it has been, just COVID, and that’s it, and unfortunately, […] if you are walking and talking and back at work […] you are not a priority [compared] to somebody who’s had a full stroke (Emma).

Emma’s comparison to stroke survivors she considered to be affected more severely was a common theme. Some participants expressed reluctance to access services they felt were meant for people with greater stroke severity:

If I were doing this for somebody else, I would’ve been a lot more stroppy about it, but because it was me I was thinking, it’s just been a small stroke, I’m alright, I’m making a fuss (Graham).

This was an example of a common feature of minimising the impact of stroke and need for support.

Participants who received ESSVR described how their RETAKE OT had the power to liaise with other healthcare providers:

Because at the time of COVID, you could not get an appointment to see your doctor […] [RETAKE OT] contacted my doctor and finally got an appointment. So, where I could not, she could because she was a medical professional (Nick).

#### 3.4.2 Post-stroke anxiety

The disruption in service provision and quality led some participants to feel they were not getting information about their stroke and their rehabilitation. Sometimes this led to, or exacerbated, feelings of anxiety:

If I get a bit dehydrated and got a bit of headache I’m thinking, ‘Oh I’m having another stroke’, do you know what I mean? it’s just in your mind all the time (Chris).

I get pains in my head and you are like […] “Oh my God, am I having another stroke?” But because I had some therapy […] I just talk myself into that every little thing was not going to be stroke-related (Tess).

Unlike Chris, Tess appears to have had ‘some therapy’ which gave her coping strategies. For some participants, the psychological impact of the stroke was felt most acutely when being discharged from community health services:

I know if I was in a much worse position, they would’ve stuck with me. I think the biggest thing for me is the anxiety of when a team’s finished it feels as if you are facing this on your own (Graham).

This anxiety seemed to stem from the information (or lack of) that they received in hospital and thereafter about how stroke might affect them. One participant used the interview to seek information:

Can I just ask you something? You might not know the answer but the only thing that worries me really is, is there any follow-up at all, medically? Are you supposed to, or is it just that—I mean, I do not really want any more bad news because I think it possibly might have—It was a very small clot (Wendy).

Wendy seemed to be struggling to make sense of her situation without adequate advice or support.

Sometimes a lack of clear guidance led to potentially ill-advised RTW choices:

In the hospital, I said what about going back to work? and [the doctor] said, “just take it easy.” I did not really know what that meant, so I thought, well I cannot drive for 4 weeks, I’ll take 4 weeks off. If they’d said to me, you cannot drive for 10 weeks, I’d have probably had 10 weeks off work (Chris).

In contrast, one participant who received ESSVR described how his [RETAKE] OT helped him RTW at an appropriate pace:

Without [RETAKE OT], I would’ve tried to get back to work far too quickly. I would never have entertained the idea of doing an hour or two hours a week. I just could not have factored that in (Graham).

Another ESSVR recipient described how his OT continued to help him to understand the impact of stroke after supporting him to RTW:

Getting back to work is one thing […] but it’s been really helpful to have [OT] to talk to and say, “I’ve been really struggling with this, is it normal?” […] drawing on her professional understanding of stroke […] without her I would not have access to that information and understanding which I’ve found really helpful in terms of my rehab (Liam).

These reported experiences indicate the importance of ESSVR in helping stroke survivors come to terms with the disruption experienced post-stroke, particularly during the pandemic.

### 3.5 Theme 2. Disruption to working patterns and practices

Government directives to work from home (where possible) during the pandemic meant that many employers had to make changes to working patterns:

Prior to COVID-19, we were not really accustomed to working from home. The nature of our business […] you need people on site […] but with COVID-19 we had to really look into that and start to have hybrid working policies (Employer D, Manufacturer).

It seems that this employer’s business was not deemed ‘essential’ (unlike healthcare and education for example) and consequently had to close their premises during the pandemic. Whilst disruptive for businesses, furlough presented opportunities for some people to improve their work/life balance and pursue hobbies, as this stroke survivor described:

I might go out [cycling] in a morning before work. I’d finish work at lunchtime on furlough, I’d go out again in the afternoon, you know, I might do the school run, then I’d go out [cycling] again at six o’clock at night (Chris).

One stroke survivor described how his work patterns had become more manageable due to changes introduced during the pandemic:

It started with COVID […] everyone was working from home and for me that’s carried on. In my job […] we are out and about a lot and when I’m done I tend to finish up from home […] It’s much easier to manage for me [than returning to the office] (Liam).

This was an example of how workplace culture changed post-pandemic. Some companies made more radical changes, as this employer described:

[Before the pandemic] there was plenty of flexible or remote working […] During the pandemic the organization made the decision to go wholly remote working […] most of our office spaces, the leases have expired, and we have not renewed them […] we are now wholly remote working (Employer C, health-related charity).

Along with many workers, stroke survivors saw the benefits of disrupted ways of working when allowed to work remotely:

When it first started, I thought it was brilliant because I wasn’t spending £50 a day on fuel, working from home saved me a fortune! (Nick).

However, some participants, including Nick (above) found that working online exacerbated the cognitive impacts of stroke:

After an hour of Skype meetings, I’m shattered. I cannot concentrate or do anything productive; I had to go and have a lie down (Nick).

I’m a home worker anyway, so the pandemic made work more intense actually, so that was the impact for me […] [My partner] said that before the stroke I was working all day […] being a home worker, it’s very easy to just sit at the desk all day and not take proper breaks (Graham).

Recognising that remote working was potentially more intensive, some stroke survivors described how they managed the problem of post-stroke fatigue:

Personally, I found it easier to not be with people because it could be quite exhausting, but also, it’s actually quite tiring to be on screen all day as well. So, I had to make sure that the meetings had a good gap between them because I could not have done an hour’s meeting that was quite intense and then gone straight into another meeting […] I was able to make sure that I scheduled stuff so that I could actually rest in between times (Jackie).

Despite having some benefits, some participants felt that remote working reduced enjoyable social elements:

Well, we are all working from home, we do not see each other. People get married, people have babies, we do not hear about it until an email comes out 6 weeks later. [Before COVID] we’d go out once a month and have a couple drinks and a meal. We lost all that (Lynn).

The social aspect of work was also highlighted by an employer:

When we did the home working it did not necessarily seem to suit [stroke survivor] because he’s used to company and interacting with other colleagues. I sensed that he missed the presence of colleagues in the office where we tend to chat about various things […] so I think there were pros and cons of COVID-19 and working from home (Employer D, manufacturer).

Other employers described how employees supported each other:

Surprisingly, during COVID when we could not go to the office and we all moved online, the support that we felt toward each other actually increased dramatically and we have really become closer (Employer A, patient advocacy charity).

For an hour every Friday we all get onto Microsoft Teams and play a quiz […] you can grab a brew and just have a bit of non-work, a bit of social (Employer E, Civil Service).

#### 3.5.1 Self-employed participants

Changes made to working practices affected the two self-employed stroke survivors in the study differently. One participant had opened her own salon 3 months before the UK went into government-sanctioned lockdown (where residents were asked to stay at home except for certain activities, e.g., ‘essential’ work, grocery shopping, and medical care). Because this was a new business, the stroke survivor was not entitled to government financial support over lockdown. Once salons reopened, she increased her working hours to compensate for lost revenue. She believes this stressful experience potentially caused her stroke:

Anything personal care was closed down. [I was] worried about money. When you have got a family home, I’ve got kids in uni, a partner who’s not well, you know, so [when it reopened] I was doing 70 h and I think that’s what did it. Even the hospital told me off (Emma).

The other self-employed stroke survivor had a very different experience. As a legal guardian of children studying in the UK, many from Asian countries, there were pandemic-related anxieties (e.g., getting children back to their families and children being unable to return home). However, this participant was able to access government support:

It did affect me during lockdown because obviously I could not charge a whole fee, but I could charge a retainer. So, it wasn’t too bad, there was quite a lot of help for the self-employed through the government. So, […] I did not really lose any money through COVID, and I did not lose that much through my stroke. I’ve been very lucky actually (Wendy).

### 3.6 Theme 3. Disruption to domestic life

During the pandemic, people considered extremely clinically vulnerable (e.g., people with an underlying disease or health condition such as asthma, who risked severe illness if they caught coronavirus) were advised to stay at home and avoid non-essential face-to-face contact (HM Government, 2020). This ‘shielding’ advice potentially affected stroke survivors as well as their relatives:

I still stay at home. Basically, I do not want to go out because of COVID. And my partner suffers from [an inflammatory bowel disorder], so it could kill her if she caught it (Nick).

The main impact for me was that I lost my childcare [Before the pandemic] my parents would pick [child] up from school which meant I could work longer […] and we lost that because my dad’s got cancer, my mum’s got a heart condition […] we minimised contact because obviously we did not want to give [COVID] to them (Tess).

Stroke survivors with young children experienced extra pressures as childcare arrangements and schools were disrupted by the pandemic. This sometimes impacted on their ability to work:

We had a problem when schools were shut, because then I’d got the wife at home, little lad at home, […] and trying to juggle, you know, trying to do your work and trying to […] do a bit of home schooling (Chris).

One stroke survivor and their partner were classed as ‘key workers’. Consequently, their child was allowed to attend school so that they could continue working:

I work at [company], that was deemed key working, and my other half, he’s an HGV driver […] So, because we were both key workers, [my child] got a place at school’ (Tess).

During the pandemic, some stroke survivors took on extra care responsibilities of family members (adult children and vulnerable parents). Some found this enjoyable:

[My daughter] moved in, and we have a very small house but we have a shed in the garden, like an office-y thing, so they moved into that and lived with us for 6 months because the lockdown happened […] I think that’s why I enjoyed it so much, because it was the whole family together (Jackie).

Nevertheless, working from home arrangements required compromises in the use of domestic spaces. Jackie continued:

I found it much more tricky to concentrate when I’d got people around. I know that’s the same for everybody, but I think I was finding it a bit harder because the house is small and there wasn’t space […] I could escape to and be working. My husband’s got a room upstairs, he could close the door, but I was left in more public spaces, which was fine because he’s the chief money earner (Jackie).

Jackie prioritised her husband’s needs for a peaceful workspace at the expense of her own need for quiet.

### 3.7 Theme 4: disruption to identity

Stroke survivors described disruption to their identities, roles, and future plans.

#### 3.7.1 Disruption to work identity

Work represented a part of normal routine for stroke survivors; who they were, who they are now, and to their identity. One employer described their employee as:

A very proud person. For her, being in work is very, very important. So yeah, she wanted to work (Employer A, patient advocacy charity).

Some participants were anxious not to lose this part of their identity when they experienced stroke:

So when I was saying I wanted to bring my computer [into the hospital], they were like, “But you should be resting.” I was like, “Well, mentally, I feel fine. I’ve got a business to run. If I do not do anything, even if it’s just putting in place strategies to manage existing clients, I will not have a business. I’ll have nothing” (Wendy).

Along with needing to work for financial and business reasons, work seemed central to Wendy’s identity as independent, which was threatened by hospitalisation following stroke:

I’m not very good at being vulnerable, I think is probably what I already knew and what was cemented during those 7 weeks [in hospital]. So, I do not like being vulnerable. I do not like not being independent (Wendy).

All but one of the stroke survivors had RTW at the time of their interview and many of them had resumed their pre-stroke hours. For some, RTW presented a chance to reclaim a sense of pre-stroke identity. However, some (female) stroke survivors raised that their partners were unhappy with their decisions to RTW, creating tension in their domestic lives:

My other half does not—did not want me to—he still does not want me to work. And I’m like, “I have to.” You know, “I have to do that.” But then I’ve got him going, “I do not want you to work” […] and you are like, “Oh my God, I just want to go back. I just want me” (Tess).

Tess seemed frustrated by her partner’s lack of awareness of the importance of work to her sense of identity and normality:

The stroke’s taken so much away from me, it’s almost like you are going, “I want some back,” you know; “I want some of me.” And when I’m at work, I’m me. It does not matter that my leg or my arm does not work properly. My head does and I can still fulfil my job. And I’m normal, if you like (Tess).

#### 3.7.2 Return-to-work experiences

A drive to regain some normalcy, led to some participants contemplating RTW too quickly:

I thought, if I can get back to work it’s more normal. So that’s why I pushed myself to get back to work, to have a bit of normality, to see people, to talk to people on Teams or to go in when I had to go in, yeah. It was normality for me (Lynn).

For some stroke survivors the urge to return to ‘normal’ included withholding information about their stroke:

I have told some of my close clients who have known me for 32 years, they know, but not everybody (Emma).

I’ve got to be honest; I only told a few people. I was a bit embarrassed really […] I did not even apply for a disability badge for my car because I did not want the label (Wendy).

One participant resisted being labelled at work, preferring to maintain the illusion that she was unaffected by her stroke:

They did ask me did I want a DSE assessment. But I’m okay, I am okay. I’m not on crutches or anything […] we have got a lift. I do not need a special mouse because my hand is fine now. Everything’s working (Lynn).

Lynn seemed to want to distance herself from ‘other’ disabled employees:

I did not want to be treated like, you know… We’ve got a lot of people with different conditions in work and I did not want to be on the—they are all on one bank of desks and all that (Lynn).

Concern about how they were perceived was sometimes due to colleagues’ misunderstandings around stroke. However, this was not reported as a lasting impact:

I do not know if that was me, in my mind, that when I was talking to people, do you know that ‘is he alright? is he taking it all in?’ But I think now they have all just sort of forgot about it (laughs) (Chris).

One participant decided to be upfront about her stroke:

I gave people permission: ‘if you are curious, just ask’ (Jackie).

Participants who told employers and colleagues about their stroke generally felt supported. One employer appreciated their employee’s openness:

[Stroke survivor] learned to be good about sharing information, that was important (Employer A, patient advocacy charity).

Another employer described how open conversations facilitated a manageable RTW:

We made some adjustments after listening to how she would prefer to work […] we created a very clear role for her […] very structured, protected from additional stresses and pressures [.] where she can use her incredible planning skills to plan her work […] We also listened to how she wanted to RTW and how many days she wanted to do (Employer C, health-related charity).

Having honest conversations with employers helped stroke survivors to adapt to changed work identities:

They’re like, “Can you do this?” and if I say no, there’s no kickback or anything […] They’re like, “That’s fine, we’ll find someone else to do it.” So, I’ve been extremely lucky at work […] there was never any pressure; it was all on my terms, it was like, “you do what you want to do” (Tess).

As the above quotes reveal, successful RTW was aided by having understanding employers. However, some employers had no procedures for supporting someone to RTW after stroke:

They [company] tended to have a younger workforce demographic. So when they had an individual off sick long-term they did not have [an] established process; they were not sure how to best manage the situation (Employer B, HR consultant).

In such cases, the RTW was often led by the employee:

It was very much (stroke survivor) who took the lead on finding out about things that might be able to support her. [As an organisation] we were expecting people to tell us what they needed rather than proactively working with them to identify those needs (Employer C, health-related charity).

In the above case, the stroke survivor was subsequently instrumental in developing policies and procedures for her company to follow.

ESSVR participants benefitted from specialist VR support:

We met my line manager before I went back to work and [RETAKE OT] laid out what the [stroke] impacts were and what I’d probably need […] We came up with a plan for my RTW together (Liam).

On the advice of the OT, Liam’s employer agreed an extended phased return and supported him to work from home to manage noise issues. The value of having expert knowledge to facilitate RTW conversations was highlighted by employer B:

I’ve always been a big supporter of allied health professions supporting people’s long-term RTW because they have those skills around rehabilitation of certain conditions. And that’s specialist knowledge that GPs often do not have (Employer B, HR consultant).

Another employer expressed appreciation for the RETAKE OT’s support:

For the employer, having trust in the OT that they really know what’s happening with [the stroke survivor] is very important, I think it was good. We were able to get in touch with [OT] anytime we wanted. The doors were open (Employer A patient advocacy charity).

#### 3.7.3 No RTW

In some cases, RTW was not possible during the pandemic where, for example, the role involved overseas travel. Also, some roles were not compatible with stroke-survivors’ abilities:

He was chomping at the bit to get back to work […] but the [occupational health] report said he’s unfit to work in any capacity. Even though he feels physically okay, he’s still having cognitive issues. Fatigue is a big thing, and slight memory loss […] We did look to see if we could put him into any other roles, but there wasn’t any (Employer E, civil service).

The only stroke survivor in this study who did not RTW explained that although he valued and enjoyed his work, he worried that his capability and reliability could no longer be taken for granted. Consequently, he decided to take advice to retire rather than risk issues arising due to the impacts of stroke:

I think [medical retirement] is the best decision for me, because […] if I miss something people’s safety is being affected. And I would not be able to say, “I’m a hundred percent, I can do that.” […] Because if it goes wrong and I miss something, which I’m likely to, it’s their life. I cannot mess about with people’s lives.’ (Nick).

Despite acknowledging the necessity of retirement, Nick reflected on how stroke had disrupted the way he had envisaged his future:

Your life’s all mapped out […] you are going to do this, you are going to do that, another 4, 5 years and you are going to look at early retirement, and then all of a sudden you have a stroke, and […] it throws all your plans out the window. Hey, you have got to live with it and move forward (Nick).

The importance of work was evident in Nick’s post-retirement plans:

I’m looking at voluntary work in the community […] There’s a men’s club where people can use their experience to fix things, bikes and so on, and just do that sort of thing. […] But it’s frustrating that […] you have got to suddenly stop your life but not sit around […] you have got to put a new structure into your life and move forward (Nick).

Nick’s plan to re-create his work identity in a voluntary capacity illustrates the need for support to explore alternative options when RTW at pre-stroke roles are untenable.

Despite making considerable progress in their recovery, many participants felt that their work identities had not completely returned to what they were pre-stroke. Impacts of stroke sometimes entailed making changes to the way they worked:

I’m on 4 days at the minute. I do not think I’ll increase it to five because I’ve had a warning […] I do not want another stroke (Tess).

One stroke survivor valued having support to accept, and adapt to, post-stroke abilities following disruption:

The real skill that [RETAKE OT] gave me was to enable me to tell myself that actually what I was doing was fine, because it felt to me so much less than what I was doing before. But actually, it is fine, and nothing’s kind of fallen apart. You know, I was probably doing far too much. […] I have a far nicer quality of life now, and I’m also able to work effectively (Jackie).

For these participants, stroke was a catalyst for changing their work/life balance.

#### 3.7.4 Disruption to domestic identity

In addition to paid work, there was an emphasis on ‘getting back to’ housework for some stroke survivors, especially female participants:

Coming back [from the hospital], even just walking into the house, and I think I swept the kitchen the next day, I just wanted to go and sit down afterwards. It was ridiculous (Jackie).

Jackie felt the impact of her stroke most acutely when trying resume her domestic role.

Partners expressed concern over stroke survivors performing tasks such as cleaning and gardening:

In the beginning my husband was obviously worried and would not let me do this, would not let me do that, and I was like, God! He was more frightened than I was (Lynn).

Concerns that their families were being over-protective were more commonly expressed by female participants. One participant revealed that this was why she had avoided telling some family members about her stroke:

I do not want, especially my dad and my older sister, she’s got problems, so I do not want to worry her. Because she would not leave me alone, she’ll be at the door every day […] it’d be more stressful telling her and my dad than not telling them at all, and then I could get on my life. Because otherwise it’ll be, ‘oh my god, why’re you going to work, why’re you driving?’ you know (Emma).

Some stroke survivors struggled with disruption to their identities as ‘carers’ where the people they normally cared for, were, in some cases, taking care of them. They described discomfort about upsetting their families:

My family were obviously shocked. [My daughter] came down, obviously it was COVID, we were limited where we could go […] she did the ironing in between crying. And that got me down because she was crying all the time. Because people always think the worst, do not they? But they were good, my family. My husband was marvellous (Lynn).

There was a reported need to manage the stroke impact to reassure family:

I think I needed to grieve, if you like, grieve for my old life […] I’m appreciative that I’m still here but sometimes you do get frustrated, like, I’ve got a 12-year-old [child] and I cannot do the stuff that I could do with him before […] But then I think actually, I’m still here for him so, you know, you have got to talk yourself round that (Tess).

Despite her limitations, Tess recognised the continuity of her parental identity, albeit in new ways.

#### 3.7.5 Disruption to identity as ‘fit and healthy’

When describing the impact of their stroke, many participants seemed to seek to minimise disruption by couching the effects of stroke in positive language, including feeling ‘lucky’:

I was very, very fortunate with my stroke in as much as it affected my speech and thought processes. I have to stop and think about what I’m saying, then once I’ve thought about it, I can say it out loud. I’m lucky […] I have not lost my speech, it’s just very measured now what I say. And I get fatigued very, very quickly (Nick).

Participants described how stroke had disrupted their idea of themselves as relatively healthy:

It’s a big change […] I mean the only other time I’ve been in hospital is when I had my son […] I never go to the doctor’s […] I’ve never been a poorly person (Tess).

For some stroke survivors, it became important to find out why stroke had happened:

I think it helped [stroke survivor] having an explanation of what caused it in the first place because in the immediate aftermath he was a bit puzzled […] he’s relatively active and had no major prior medical conditions […] He was given some tips on how to reduce his cholesterol and that gave him a bit of confidence to say ‘yes I can get back into day-to-day work because I have a bit more information about what led me there’ (Employer D, manufacturer).

Many stroke survivors recognised the need to change their lifestyle and adapt to post-stroke abilities:

I just have to be really careful about what I’m doing, making sure I get enough sleep, that kind of thing. […] Before my stroke, I would be bouncing out of bed really early in the morning and doing yoga practise and facing the day with lists and things to do […] I cannot do that anymore. I’m much slower about getting up in the morning. I can do that stuff, but I just do it differently (Jackie).

Participants who previously considered themselves to be fit and active described trying to re-establish this aspect of their lives whilst also minimising chances of having another stroke:

I did not push it too hard on my bike, but now I’m back, not doing as many miles but fitness-wise, you know, pushing hard […] but just reined a lot of beer in. I do not go daft (Chris).

Returning to cycling represented reclaiming an important part of Chris’s identity but he also recognised that his heavy-drinking days were over.

## 4 Discussion

This study explored stroke survivors’ and employers’ RTW experiences during the pandemic using NPT (May et al., 2009). The aim was to understand the impact of the pandemic and the furlough scheme on stroke survivors’ work ability and RTW support at that time through the lens of biographical disruption (Bury, 1982).

In common with most of the general population, participants experienced the pandemic as disruptive to almost every area of their lives. However, for stroke survivors, the pandemic added extra layers of disruption.

For many participants, this was their first experience of stroke, and being of working age, it represented not only a ‘profound diversion from their life trajectory of building their career’ (Ibrahim et al., 2024, p. 5) but also disrupted their idea of themselves as fit and healthy. Similar disruption to identities as relatively healthy individuals was reported in previous studies of stroke survivors aged below 55 (Kuluski et al., 2014). This contrasts with older stroke survivors who often consider stroke to be part of the ageing process and consequently less unexpected (Faircloth et al., 2004; Pound et al., 1998).

In our study, the unexpected experience of stroke led to many participants questioning why it had happened (meaning as consequence) (Bury, 1982). Some attributed stroke to their stressful job, such as the recently self-employed participant who tried to compensate for lost earnings due to initial lockdowns. Others struggled with making sense of the changes in their work and social lives following stroke (‘sense making’; Gallacher et al., 2013), and experienced difficulties mobilising the necessary resources (e.g., information and support) that might minimise this disruption (Bury, 1982). GP appointments were scarce, and most consultations were conducted by telephone. This created barriers to accessing information and advice about the impact of stroke, the likelihood of RTW, and ways to adapt to post-stroke abilities. Our findings therefore highlight the significance of interacting with others who have condition-specific knowledge when experiencing uncertainty about the appropriateness of RTW post-stroke (Gallacher et al., 2013), in this case VR-trained OTs, who are often well-placed to provide RTW support to patients and employers (Drummond and Smyth, 2023).

For some participants, lack of information about stroke affected their mental health, with symptoms such as headaches being (mis)interpreted as stroke recurrence. Although health anxiety is common amongst stroke survivors, Rumrill et al. (2022) found that mental health difficulties emerged or were exacerbated during the pandemic. Similarly, Ahmed et al. (2020) reported higher rates of post-stroke anxiety during the pandemic which they attributed to social deprivation and lack of rehabilitation services. This was evidenced in our findings where post-stroke anxiety was heightened by difficulties accessing information and support, and/or reluctance to further burden the NHS. A study of people with chronic disease reported similar reluctance to access health services during the pandemic, even though they risked potential harm by not seeking care if needed (Luck et al., 2022).

Some participants tried to see the lack of support in a positive light, i.e., that their stroke was insufficiently severe to require long-term support. Indeed, there were frequent uses of positive language, including multiple references to feeling ‘lucky’ in the stroke survivor interviews (but notably, not employer interviews). As well as considering themselves ‘lucky’ in respect of the support they received from families, RETAKE OTs, employers, and colleagues, stroke survivors frequently suggested that they were ‘lucky’ compared to people who they considered to be more severely impacted by stroke. Wills (1981, p. 245) refers to this as downward comparison where ‘persons experiencing negative affect can enhance their subjective well-being through comparison with a less fortunate other’. Downward comparison sometimes led to stroke survivors considering themselves less deserving of medical attention and support, and consequently not seeking it. An exception was ESSVR participants who seemed more comfortable to accept support and information from their OT. This may be because interacting with others with stroke-specific knowledge and experience was integral to the ESSVR intervention, and consequently their use of this resource was legitimised (Gallacher et al., 2013; May et al., 2009).

The pandemic also disrupted businesses. Some were forced to close, leading to negative financial and social implications for employees and owners. Participants who had recently become self-employed were particularly affected as they were ineligible for the government’s self-employment Income Support Scheme (Gov.UK, 2020b).

Some participants received financial recompense through the furlough scheme which paid a percentage of wages for people unable to work. In this study, stroke survivors considered furlough beneficial for allowing them to enact management strategies for dealing with the disruption caused by stroke (Gallacher et al., 2013). For instance they appreciated having more time to physically recover from stroke, increased opportunities for exercise, and valuable family time, and for some, to work from home for a period of time. Nevertheless, our findings echo research suggesting that furlough and reduced working hours had different effects on men’s and women’s mental health. Wang et al. (2020) argue that women were more adversely affected mentally by the pandemic, due to role conflict. Role conflict theory suggests that overlapping social roles and responsibilities make it difficult to comply fully with either work or family commitments (Greenhaus and Beutell, 1985). In our data, more women than men described competing roles when working from home whilst also supporting family members physically, financially, and emotionally. The more frequent references to housework and childcare made by female participants is consistent with figures indicating that women continued to bear the brunt of domestic caring responsibilities during the pandemic (ONS.gov.uk) suggesting a return to traditional normative familial patterns and ‘creating the risk of a “re-traditionalization” of gender roles (Morero-Mínguez and Ortega-Gaspar, 2022; International Labour Organisation, 2021).

Stroke added an additional layer of disruption to these domestic roles, with some female participants struggling to be the person being cared for, rather than the carer. In some cases, this included ‘protecting’ family members by not disclosing the extent to which stroke had affected them, or even not telling them at all. Faircloth et al. (2004) argue that ‘bracketing off’ the impact of stroke helps people to maintain a coherent sense of self and minimise disruption. Our findings suggest that the pandemic facilitated non-disclosure, as face-to-face contact was seriously curtailed. And although this facilitated a biographical flow (Faircloth et al., 2004), failing to reveal the extent of the impact of stroke sometimes resulted in stroke survivors not receiving appropriate support, including workplace adaptations. The absence of appropriate management strategies to adapt to post-stroke abilities has potential implications for physical and mental health, and chances of sustained RTW. Conversely, stroke survivors who openly discussed the impact of stroke and their needs through interactions with others, including family members and employers, had more positive RTW experiences, particularly when supported by RETAKE OTs (Gallacher et al., 2013).

Findings echo previous research indicating disruption to homelife resulting from stroke (Kuluski et al., 2014). This was compounded in the pandemic by Government directives to work from home, leading to an increase in remote working from 5% in 2019 to 37% in 2020 (Bank of England, 2021). This disrupted established ways of working and sometimes caused tensions around finding space to work within the home. However, it was reported to be beneficial for enabling stroke survivors to RTW during the pandemic. Avoiding commuting benefitted those unable to drive physically or through lack of a driving licence. Furthermore, working flexibly enabled stroke survivors to enact management strategies such as planning and using their social and work time more effectively and subsequently reducing stress and post-stroke fatigue (a common barrier to RTW), and improving work/life balance. Similar benefits of working from home have been reported in studies of non-disabled people (Office for National Statistics, 2022; Trusson et al., 2023).

Importantly, working from home during the pandemic has been shown to be effective and become a more acceptable way of working. This change in workplace culture has positive implications for people with disabilities who might be enabled to work remotely, particularly as more businesses move online. Saia et al. (2021) argue that the pandemic may thus serve the broader equal opportunities agenda by providing new homeworking opportunities. However, it is important to note that working from home was not possible for some participants including those whose role was unsuitable for remote working and those whose post-stroke impairments made it difficult to use technology. Consequently, our findings support research suggesting a higher likelihood of RTW for stroke survivors in ‘white collar’ jobs (Edwards et al., 2018; Ibrahim et al., 2024) post-pandemic.

Findings suggest that many participants considered returning to their usual workplace as a significant step to returning to ‘normality’ following the disruption of stroke. Furthermore, female participants seemed particularly keen to restore social contacts lost in the pandemic which resonates with Burn et al.’s (2022) research showing a higher prevalence of depression symptoms amongst women working from home compared to those travelling to a workplace. These findings highlight the benefits of ‘going out to’ work to restore routine, improve social contact and provide a degree of identity continuity (Wolfenden and Grace, 2015). However, this option was reduced during the pandemic and may no longer be achievable due to an increased trend towards more remote, or hybrid working (Office for National Statistics, 2022). This was evidenced in our study where one employer described moving to working online entirely, with no physical offices post-pandemic.

Equally, some stroke survivor participants re-examined their plans for the future post-stroke and post-pandemic through ‘appraisal work’ (Gallacher et al., 2013). This included reflecting on their priorities, with some participants taking actions to improve their work-life balance and reduce stress, thus reconfiguring their biographical future (May et al., 2009; Trusson et al., 2021). Where RTW at the same job was not possible, participants explored alternatives, e.g., volunteering, indicating the importance of work for biographical continuity.

### 4.1 Limitations

This study is limited by relatively low numbers of participants. Although this limited the generalisability of the findings, we feel that the sample was sufficiently diverse to represent a range of viewpoints and experiences and develop a robust and theoretically informed explanation of participants’ experiences. For example, we achieved our aim of including at least 50% female participants to address the gender imbalance in the larger RETAKE study. There was also diversity in terms of employing organisations and employment experiences. Some participants were furloughed, some worked from home, some were considered ‘key workers’, whilst others were employed in industries particularly affected by the pandemic. In addition, the two self-employed participants had contrasting experiences during the pandemic in terms of government support.

As expected, employers proved particularly difficult to recruit (Coole et al., 2013). However, mitigating measures ensured that we gathered the perspectives of five diverse employers about working during the pandemic and supporting stroke survivors to RTW.

## 5 Conclusion

Multiple aspects of daily life were disrupted by the pandemic, but it was particularly disruptive for people experiencing stroke during that time. Stroke disrupts people’s previous security and identity as healthy. At the same time, services that should have helped them to accept and adapt to life post-stroke, were themselves disrupted. Lacking information and support sometimes led to health anxiety, which was often unaddressed due to reluctance to add to NHS pressures.

The benefits of ESSVR when dealing with a disruptive health experience amid a pandemic were evident as RETAKE OTs helped stroke survivors and employers to adjust and adapt to post-stroke abilities. Furthermore, post-pandemic working culture changes (including greater acceptability of remote working) may benefit stroke survivors in future when aiming to RTW. However, there are also social and mental wellbeing benefits to returning to the workplace. Overall, RTW (either pre-stroke employment or an alternative role) may mitigate biographical disruption for working-aged people who experience stroke.

## Data Availability

The datasets presented in this article are not readily available because data supporting this work are available on reasonable request. All requests will be reviewed by relevant stakeholders, based on the principles of a controlled access approach. Requests to access the datasets should be directed to CTRU-DataAccess@leeds.ac.uk.
