# Supplementary material for: Exploring stroke survivor and employer experiences of disruption within the RETurn to work After stroKE (RETAKE) trial during the COVID-19 pandemic
Source: Front Sociol. 2025 Jul 2;10:1434353. doi: 10.3389/fsoc.2025.1434353 (PMC12263687; doi:10.3389/fsoc.2025.1434353)
Supplement: Supplementary file 1 [file Supplementary_file_1.docx]

Appendix 1: Interview topic guides

Stroke Survivor Interview Topic Guide

Opening/Introduction: Thanks for participating in the study and for giving up this time.

Reminder of the focus of the study: We are interested in finding out about your stroke, and any support you received to return to work. We would want to understand the impact the COVID-19 pandemic has on you and your ability to work.

1. Can you tell me a little about when your stroke happened and how your stroke affected you? (NPT construct ‘sense making’)

· *Physically, emotionally, socially, family life, etc*

2. Can you tell me about your work situation before you had the stroke?

· *Part-time? Full-time? Voluntary? Redundancy?*

· *Responsibilities/Job role*

· *Relationships (good relationship with employer?)*

3. Prior to the Covid-19 pandemic, had you returned to work?

*If no*

· *Can you tell me why you hadn’t returned to work?*

· *Did anyone suggest any alternative options (e.g., new job role)?*

*If yes*

· *How long have you been back at work?*

· *Phased return, part time, full time? Paid? Voluntary?*

· *How has work changed since your stroke? Did you have any adjustments or adaptations put in place? (e.g., changes to role/responsibilities, use of specialist equipment, use of energy conservation techniques, changes in how they travel to work)*

· *Has anything gone particularly well since you had been back at work?*

· *Have you experienced any difficulties since you had been back at work?*

· *Have you received any support since returning to work? (Health professional, employer example line manager, human resources, occupational health, GP, family, etc)*

·  *If yes*

o *What did they do to support you? (e.g., give information, advice, suggestions)*

o *(if discussing health professional) Did they provide any support to your family or employer?*

o *Is there anything else they could have done to help you or your family/employer?*

6. Had/have you discussed your return with anyone in your workplace (e.g., line manager, HR)? (NPT construct: Interacting with others)

*If yes*

*a.* *Did they stay in touch with you? Were you able to contact them?*

*b.* *What happened when you discussed it with them?*

*c.* *Were they supportive / knowledgeable about stroke?*

*d.* *Did they agree with- or make any suggestions for your return to work?*

*a.* *Was there anything you or someone else (e.g., OT) needed to negotiate with them?*

7. How has the Covid-19 pandemic affected your return to work? (NPT Construct: Enacting Management Strategies)

· *Contact/support from others (health professionals, employer)*

· *Access to resources/equipment (e.g., computer)*

· *Transport to/from workplace*

· *Returning to work sooner than ideal (e.g., essential worker, needed money)*

· *Started new temporary role (e.g., supermarket, NHS volunteer, childcare)*

· *Reduced hours, or not able to work (graded return postponed, furloughed, shielding/self-isolating, made redundant)*

8. Have you experienced any unexpected benefits in how you’ve been treated during the Covid-19 pandemic?

· *More regular contact with health professionals/employers*

· *Provision of support (e.g., information, advice, equipment, financial)*

· *Reduction in fatigue (e.g., through not having to travel as much, etc)*

9. Was returning or not returning to work the right decision for you? (NPT construct: Appraisal work)

· *E.g., retirement, hobbies, financial implications*

10. What are your plans for the future? (If not back at work - does this include going back to work?

**Employer Interview Topic Guide**

1. Could you tell me a little bit about your organisation? For example, the size, type, nature of the organisation, etc.
2. How would you describe the culture of your organisation in terms of supporting people to return to work after long-term sickness absence?
3. Have you had any experience of supporting someone with a serious health condition to return to work?
4. How much background knowledge did you have around stroke?
5. Prior to [Stroke Survivor] speaking with you recently about this interview, did you know that [Stroke Survivor] was involved in a research study?
6. Did anything get in the way of you helping [Stroke Survivor] get back to work? For example, a lack of resources or a lack of knowledge, etc?
7. Just thinking how the pandemic has affected your business. What kind of overall impact has the pandemic had on your business and conduct, if any?
8. Did your company use the furlough scheme?
9. Do you think that the pandemic created problems in [Stroke Survivor]’s return to work process?
10. Is there anything else you’d like to add or comment on around the pandemic or the challenges of supporting people to return to work after a stroke?
